# P-276. Temporal Trends in Racial Disparities of HIV Linkage-to-Care within Emergency Department-Based Testing Programs at a Community Healthcare System, 2018–2024

**DOI:** 10.1093/ofid/ofaf695.497

**Published:** 2026-01-11

**Authors:** Jianli Niu, Paula A Eckardt, Elizabeth Thibodeau, Sheila Montalvo, Eric Boccio

**Affiliations:** Memorial Healthcare System, Hollywood, Florida; Memorial Healthcare System, Hollywood, Florida; Memorial Healthcare System, Hollywood, Florida; Memorial Hospital System, Cooper City, FL; Memorial healthcare System, Hollywood, Florida

## Abstract

**Background:**

Emergency department (ED)-based HIV testing programs have been widely implemented across the United States. Linkage-to-care rates among newly diagnosed individuals with HIV vary widely and many remain suboptimal. We aim to evaluate the overall linkage-to-care rate and explore temporal trends in racial disparities of linkage-to-care from 2018 to 2024 within an ED-based HIV testing program at a community healthcare system in South Florida.

**Figure 1.**
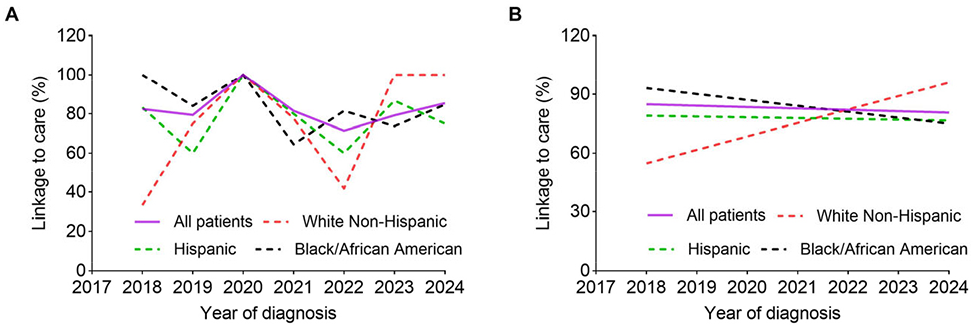
Trends in linkage-to-care rates by racial group within the Memorial Healthcare System ED-based HIV testing program from 2018 to 2024. (A) Linkage-to-care rates (%) across racial groups by year within the Memorial Healthcare System ED-based HIV testing program from 2018 to 2024. (B) Temporal trends in linkage-to-care rates (%) across racial groups by year within the Memorial Healthcare System ED-based HIV testing program from 2018 to 2024, assayed using Joinpoint regression analysis.

**Methods:**

This retrospective analysis utilized data from the Memorial Healthcare System’s ED-based HIV screening program (2018–2024) to quantify linkage-to-care rates and assess racial disparities in care linkage. Annual percentage change (APC) was measured using Joinpoint regression models to describe the temporal trends in HIV linkage-to-care rates from July 2018, when the screening program was initiated, to December 2024, based on the most recent data available at the time of analysis.

**Results:**

From 2018 to 2024, the average HIV linkage-to-care rate of the ED-based HIV testing program was 82.8%. The average linkage-to-care rates were 84.1% among Black/African American individuals, 75.4% among non-Hispanic White individuals, and 77.9% among Hispanic individuals, with no statistically significant differences between races (p = 0.241) (Figure 1A). When using APC as an outcome, the temporal trend of the overall HIV linkage-to-care rate was stable from 2018 to 2024, with an APC of -0.32% (95% confidence interval [CI], -7.91 to 7.97; p = 0.935). There were no statistically significant changes in linkage-to-care rates across races from 2018 to 2024, with an APC of -3.37% (95% CI, -9.95 to 3.53; p = 0.345) for Black/African American individuals, 11.31% (95% CI, -5.05 to 30.5; p = 0.209) for non-Hispanic White individuals, and -0.32% (95% CI, -7.91 to 7.97; p = 0.935) for Hispanic individuals, respectively (Figure 1B).

**Conclusion:**

Our findings suggest that linkage-to-care rates within an ED-based HIV testing program in South Florida have remained suboptimal over the years, and no significant racial disparities in care linkage were revealed. Innovative strategies to improve linkage-to-care outcomes for all patients should be developed and integrated into ED-based HIV testing initiatives to enhance program effectiveness and improve patient outcomes.

**Disclosures:**

All Authors: No reported disclosures

